# PGC-1α induced mitochondrial biogenesis in stromal cells underpins mitochondrial transfer to melanoma

**DOI:** 10.1038/s41416-022-01783-w

**Published:** 2022-03-26

**Authors:** Prakrit R. Kumar, Mona Saad, Charlotte Hellmich, Jayna J. Mistry, Jamie A. Moore, Shannon Conway, Christopher J. Morris, Kristian M. Bowles, Marc D. Moncrieff, Stuart A. Rushworth

**Affiliations:** 1 Norwich Medical School, University of East Anglia, Norwich Research Park, Norwich, NR4 7UQ UK; 2 Department of Plastic and Reconstructive Surgery, Norfolk and Norwich University Hospitals NHS Trust, Colney Lane, Norwich, NR4 7UY UK; 3 Department of Haematology, Norfolk and Norwich University Hospitals NHS Trust, Colney Lane, Norwich, NR4 7UY UK; 4 Earlham Institute, Norwich Research Park, Norwich, NR4 7UH UK; 5 School of Pharmacy, University of East Anglia, Norwich Research Park, Norwich, NR4 7TJ UK

**Keywords:** Cancer, Scientific community

## Abstract

**Introduction:**

Progress in the knowledge of metabolic interactions between cancer and its microenvironment is ongoing and may lead to novel therapeutic approaches. Until recently, melanoma was considered a glycolytic tumour due to mutations in mitochondrial-DNA, however, these malignant cells can regain OXPHOS capacity via the transfer of mitochondrial-DNA, a process that supports their proliferation in-vitro and in-vivo. Here we study how melanoma cells acquire mitochondria and how this process is facilitated from the tumour microenvironment.

**Methods:**

Primary melanoma cells, and MSCs derived from patients were obtained. Genes’ expression and DNA quantification was analysed using Real-time PCR. MSC migration, melanoma proliferation and tumour volume, in a xenograft subcutaneous mouse model, were monitored through bioluminescent live animal imaging.

**Results:**

Human melanoma cells attract bone marrow-derived stromal cells (MSCs) to the primary tumour site where they stimulate mitochondrial biogenesis in the MSCs through upregulation of PGC1a. Mitochondria are transferred to the melanoma cells via direct contact with the MSCs. Moreover, inhibition of MSC-derived PGC1a was able to prevent mitochondrial transfer and improve NSG melanoma mouse tumour burden.

**Conclusion:**

MSC mitochondrial biogenesis stimulated by melanoma cells is prerequisite for mitochondrial transfer and subsequent tumour growth, where targeting this pathway may provide an effective novel therapeutic approach in melanoma.

## Introduction

Melanoma is the most aggressive, deadly form of skin cancer [1], the incidence of which is among the fastest growing cancers world-wide [2], accounting for 62,000 deaths worldwide [3]. Despite only accounting for 5% of skin cancer cases, it is the main cause of deaths in the world of skin cancer [2]. Given the rising global incidence rates [4], it is envisaged that the number of deaths worldwide are predicted to increase 175% by 2040 [3] and therefore further research is required to understand the complexity of this disease. Better outcomes can be realised through new therapies developed from an improved understanding of the biology of the disease.

Primary cutaneous melanoma comprises a distinctly heterogeneous population of both cancerous and non-cancerous cells [5, 6], including fibroblasts, adipocytes and other niche cells such as mesenchymal stromal cells (MSCs), which make up the extracellular matrix, endothelial cells of the microvasculature, and immune cells [5–7]. In addition to the cellular component of the tumour microenvironment (TME), the non-cellular component consists of several growth factors, chemokines and cytokines [8]. The tumour microenvironment (TME), both the cellular and non-cellular components, has not only been shown as a hallmark of cancer but has been envisaged as a prerequisite for tumour invasion, metastasis and angiogenesis [9–11]. Further, melanoma cells can manipulate the close association between themselves and the TME to facilitate tumour progression [12, 13], evasion of apoptosis and thereby confer chemoresistance [14].

Until recently, melanoma was considered a glycolytic tumour [15, 16], with the expression of glycolytic programs strongly correlating with hypoxia within the malignancy. However, recent research has recently highlighted the importance of oxidative phosphorylation (OXPHOS) in melanoma as well [17, 18], where both OXPHOS and glycolysis both play a significant role in melanoma metabolism [19, 20]. Moreover, recent reports have also shown that the transfer of mitochondrial DNA (mtDNA) from MSCs to melanoma tumour cells plays a key role in the metabolic output of melanoma cells, as cells regain OXPHOS capacity even in the presence of mitochondrial mutations [14, 15]. In addition, respiration recovery gained by mtDNA transfer, with fully assembled supercomplexes and respirasomes, rescued the melanoma cells from apoptosis and modulated chemoresistance, ultimately contributing to tumorigenesis [21–23].

Previously, we and others have shown that whole mitochondria can transfer from bone marrow stromal cells (BMSCs) to leukaemia blasts to enhance their proliferation in vitro and in vivo through a mechanism that increases OXPHOS in the leukaemic blasts [24–26]. Moreover, we have also observed that non-malignant human CD34+ hematopoietic progenitor cells (HPCs) can acquire mitochondria from BMSCs under infection-induced stress [27]. Also the MSC-derived from the bone marrow, have been shown to donate their mitochondria to lung epithelial cells, preventing acute lung injury [28], leading us to hypothesise that the BMSCs migrate to the site of injury or tumour to donate their mitochondria to aid recovery or enhance tumour proliferation. However, beyond the stimulation of reactive oxygen species in the TME, the mechanisms controlling mitochondrial transfer from MSC to tumour cells have yet to be elucidated.

In the present study, we sought to determine if BMSC support the proliferation of melanoma by transferring their mitochondria to melanoma cells. We also evaluated the mechanisms through which this occurs and determined if blocking mitochondrial transfer inhibits tumour burden. Together, this study will help increase our understanding of the pathophysiology of melanoma progression and metastasis and may help to develop new therapeutic interventions.

## Methods

### Materials

All antibodies (Human CD31, CD45, CD146, CD166, CD90, CD74, CD105) were purchased from Miltenyi Biotech (Auburn, CA, USA). All dyes (MitoTracker Green FM (MTG) and Tetramethylrhodamine (TMRM) stain) were bought from ThermoFisher (Waltham, MA, USA). All other reagents were purchased from Sigma-Aldrich (St Louis MO, USA), unless stated explicitly below.

### Human cell isolation

#### Melanoma

Samples of freshly harvested metastatic melanoma tumour were obtained with the patients’ prior informed consent according to our institutional biorepository protocol. The University of East Anglia’s (UEA) biorepository/tissue bank is approved by the UK Health Research Authority (HRA). UEA’s Faculty of Medicine and Health Sciences Research Ethics Committee gave approval for all other aspects of this study [Reference Number: 164]. Melanoma was isolated according to by Leelatian et al.’s protocol [29] that produced the largest viable cell yield with minimum incubation time (Supplementary Fig 2A). The FACSCanto II flow cytometer (BD, Franklin Lakes, NJ, USA) was used to confirm expression of specific markers on cells. This confirmed expression of melanoma markers (CD45−, CD31−, CD146+ and CD166+) [30], with a minimum 95% purity of melanoma cells (Supplementary Fig. 2B). Six patient melanoma samples were used, and all were confirmed to be metastatic by histology (Table 1).

**Table 1:** Patient characteristics of primary melanoma samples extracted for this study.

Specimen Number	Gender	Primary laterality	Primary site	Specimen site	Location
M#1	M	Right	Lower arm	lymph node	Right axilla
M#2	F	Left	Foot	Lymph node	Left groin
M#3	M	Left	Foot	In transit metastasis	Skin of left leg
M#4	M	Right	Foot	Lymph node	Right Groin
M#5	M	Left	Upper Back	In transit metastasis	Subcutaneous mass upper back

#### Human MSCs

Following informed consent and approval by the HRA, UK (LRCE ref07/H0310/146), patient bone marrow was obtained, and mononuclear cells were obtained via density gradient centrifugation using Histopaque and confirmed via flow cytometry, as outlined previously [31]. MSCs were isolated from BM patient samples by simple adherence to tissue culture plastics [32], with the non-adherent leucocytes removed after three days of co-culture. Flow cytometry confirmed expression of MSCs markers (CD45−, CD90+, CD73+ and CD105+) [33].

### Cell culture

Freshly harvested human melanoma cells, SKMEL28 and A375 melanoma cell lines, obtained from (ATCC (American Type Culture Collection) and ECACC (European Collection of Authenticated Cell Cultures) respectively), were maintained in Rosewell Park Memorial 1640 (RPMI), containing 10% foetal bovine serum (FBS) and 1% penicillin–streptomycin (PS) (Hyclone, Life Sciences). Human MSCs were maintained in Dulbecco’s Modified Eagle’s Medium (DMEM) with 20% FBS and 1% PS. All cells were cultured in humified culture incubator at 37 °C and were passaged they were 80% confluent. All cells were passaged a minimum of three times before use in any of the experiments.

### Apoptosis assay

Sub confluent A375-GFP (4 × 10^4^ cells) were cultured with confluent MSC (0.25 × 10^5^) for 24–72 h. At each time point cells were removed from culture by trypsin and annexin V staining was performed and expressed as % apoptotic cells. The FACSCanto II flow cytometer (BD, Franklin Lakes, NJ, USA) was used to measure annexin V staining on A375 cells.

### In vitro migration assay

In vitro migration assays were designed according to Justus et al.’s protocol [34] via transwells. Using 8.0 μm well pore sized transwells (Corning), which allowed transit of whole cells, 600 μL conditioned media from SKMEL28 melanoma and control media (RPMI) was pipetted into the bottom of the wells. Next, 1 × 10^5^ of MSCs were seeded into the top of the transwell. The cells were incubated at 37 °C for 48 h and the bottom of wells were examined for MSCs. MSCs were counted using Trypan blue exclusion assay [35].

### Real-time PCR for mitochondrial biogenesis, fusion and fission genes

MSCs, at 5×10^4^ cell concentration, and melanoma cells (SKMEL28 or harvested specimens) were seeded in top and bottom of 0.4 μm pore-sized transwells, to model interaction between the two cells types, whilst ensuring easy separation of the two cell types (melanoma and MSCs) after co-culture. After 24-h co-culture, whole-cell RNA extraction was performed according to manufacturer’s instructions, using the ReliaPrep RNA cell miniprep system (Promega, Southampton, UK). This single-stranded RNA was converted to produce double-strand complementary DNA (cDNA) and amplified, using Nugen PicoSL WTA (Redwood City, CA). RNA and cDNA yield were quantified and standardised using a NanoDrop 2000 spectrophotometer. Acceptable purity quality was agreed at an A260/280 ratio of 1.9–2.1 and 1.7–2.0 for RNA and cDNA samples. Real-time PCRs were performed with SYBR-green technology (PCR Biosystems) and corresponding genes (company). On a Roche 384-well LightCycler480, PCRs were amplified for 45 cycles (95 °C/15 s, 60 °C/10 s, 72 °C/10 s), after pre- amplification (95 °C/60 s). Using the comparative cycle threshold method [36], all analysis was performed and normalised against the housekeeping gene (GAPDH (glyceraldehyde 3-phosphate dehydrogenase)) to account for cell count. Biogenesis (master regulator PGC-1α [37, 38], PPARb, TFAM, GABPA, NRF1, TF1BM and TF2BM), fusion (MFN1, MFN2 for OMM and long term OPA1 for IMM [39–42]) and fission (DNM1L [43], FIS1 [44]) genes were evaluated for in both the MSCs.

### Mitochondrial DNA quantification

For 48 h, 5 × 10^4^ MSCs were cultured with 500 μL conditioned media (CM) from SKMEL28, A375 or melanoma cells and 500 μL RPMI media (control). This culturing was followed for all subsequent conditioned media experiments. DNA was extracted according to manufacturer’s instructions, using the GenElute DNA miniprep kit. After quantification and purity were ensured via Nanodrop outlined above, Real-Time PCR were performed with specific Taqman probes (ThermoFisher (Waltham, MA, USA)). These probes targeted human ND1 mitochondria gene and human nuclear TERT gene, on different fluorophores (VIC and FAM). On a Roche 384-well LightCycler480, PCRs were amplified for 40 cycles (95 °C/15 s, 60 °C/60 s). Using ∆∆Ct method [36], mtDNA copy numbers were calculated and normalised according to human genomic TERT.

### Mitotracker Green (MTG)/tetramethylrhodamine methyl ester (TMRM) assays

MSCs, at 5 × 10^4^ cell concentration, were cultured with conditioned media from SKMEL28, A375 or melanoma cells or control media (RPMI). After 48 h, MSCs were resuspended in 500 μL of MACS buffer (PBS + 0.5% BSA + 2 mM EDTA) and incubated with 30 nM dye (MTG/TMRM) for 10–15 min at room temperature in the dark. At end of incubation, cells were washed with MACS buffer and resuspended in 1 mL of new MACS buffer. Immediately, the cells were analysed on the CyHoy & Cube 6 flow cytometer (Sysmex-Partec, Gorlitz, Germany). A minimum of 3000 events were recorded. FlowJo software package was used for analysis, where live cell population was gated for using side scatter/forward scatter. Mean MTG and TMRM fluorescent intensity was measured to determine mitochondrial content and mitochondrial membrane potential, respectively.

### Seahorse extracellular flux assay

XFp flux cartridges were hydrated in XFp Calibrant at 37 °C overnight, prior to the experiment. MSCs were seeded at concentration of 1 × 10^4^, according to ThermoFisher’s cell density recommendations, in 180 μL of conditioned media from melanoma or control media (RPMI). This was coated with poly-D-lysine, centrifuged to ensure uniform layer of cells, and was loaded, according to manufacturer’s instructions, with Oligomycin (2 μM), FCCP (1 μM), and Rotenone (0.5 μM) into the injection ports. This experimental template was in accordance with Wave software (Seahorse Bioscience). XFp Mito Stress Kit was used to obtain OCR and ECAR values, normalised to cell number in each well.

### Lentiviral transduction

Lentiviral transduction was used to stain cell membranes of melanoma cells with GFP (Fig. 4c) and Luciferase (Fig. 3e), and MSCs’ mitochondrial membrane with mito9 (Fig. 4c). All viral stocks were stored in −80 °C and thawed on ice. A total of 1.0 μL of rLV.EF1.mCherry-Mito-9 lentivirus (Clontech Takara Bio Europe, Saint-Germain-en-Laye, France), pCDH-luciferase-T2A-mCherry (Clontech) and rLV.EF1.AcGFP-Mem9 (Clontech) were added to 1 μL of media at cell concentration of 5 × 10^4^ of MSCs or A375 cells. After 24 h, cells were washed with 1 mL of DMEM or RPMI and cultured for further week to ensure no residual virus was present. Successful transduction was ensured via detection of GFP/Luciferase/mito9 under fluorescent microscopy.

Lentiviral transduction was used to knock down (KD) PGC-1α in MSCs (Fig. 3a). PGC-1α KD shRNA stock and control ShE stock, stored at −80 °C, and thawed on ice. A total of 1.0 μL of PGC-1α KD shrNA and ShE lentivirus was transduced to MSCs plated at density of 5 × 10^4^ cells. PGC-1α KD was confirmed using qRT-PCR.

### In vitro PGC-1α KD MSC assays

PGC-1α KD MSCs and control ShE MSCs, plated at density of 5 × 10^4^ cells, were cultured with conditioned media from SKMEL28, for 48 h. After MSC isolation, MSCs were analysed for mitochondrial content (MTG) and mtDNA copy number via previous methods outlined above.

### In vitro mitochondrial transfer assay-confocal microscopy

MSCs, with their mitochondria stained with mito9 virus, and GFP-labelled A375 melanoma cells were plated onto 24-well Ibidi microscopy plate for 24 h. After fixation in 4% paraformaldehyde, the cells were stained with DAPI (ThermoFisher) for 15 min, to visualise the cells’ nuclei. Cells were washed and mounted with Fluorobright mounting media (ThermoFisher). Cells were imaged on Zeiss LSM 800 Axio Observer.Z1 confocal microscope at ×63 Oil magnification. Cells were processed and presented using ImageJ (Fig. 4c).

### Animal models

In accordance with Animal Act (Scientific Procedures) 1986, and approvals from UK Home Office and Animal Welfare and Ethics Board of the UEA, all animal work was performed. All in vivo experiments used NOD.Cg-Prkdcscid IL2rgtm1Wjl/SzJ (NSG) mice (The Jackson Laboratory, Bar Harbour, ME, USA) as human xenograft models. They were housed in individual cages, under specific pathogen-free conditions in a 12/12-h light/dark cycle with food and water provided *ad libitum*.

### In vivo PGC-1α KD assay

1 × 10^5^ luciferase labelled A375 melanoma cells were injected subcutaneously into NSG mice and were monitored for 9 days to allow engraftment. At day 9, 1 × 10^5^ PGC-1α KD MSCs were injected intravenously (IV) into the tail vein of five mice and 1 × 10^5^ control-ShE MSCs were injected IV into five mice. Following injections, mice were closely monitored for signs of bleeding and return to cages. At day 14, following 200 μL IP (Intraperitoneal) injection of D-luciferin, all mice were anaesthetised with 2–3% isofluorane/oxygen. All mice were imaged within 20 min of IP injection, using the Bruker in-Vivo Xtreme Imaging Systems (Bruker Corp., Massachusetts, USA) imager, to assess tumour growth in the test group (mice with PGC-1α KD MSCs) and control group (mice with control-ShE MSCs).

### In vivo migration assay

1 × 10^5^ luciferase labelled A375 melanoma cells were injected subcutaneously into each flank into 4 NSG mice and were monitored for 9 days to allow engraftment. At day 9, 1 × 10^5^ GFP-labelled MSCs were injected intravenously into the tail vein of two mice, with the other two control mice receiving no injection. At day 15 mice were sacrificed with increased CO2 exposure and neck dislocation. Tumours were excised out (*n* = 8) and processed by Leelatian et al.’s protocol for isolation of melanoma cells and MSCs. These isolated cells were resuspended in MACS buffer. Flow cytometry analysis, after gating for live cell population using forward and side scatter, demonstrated mean GFP fluorescence for each tumour. This quantified proportion of GFP-labelled MSCs in the tumour.

### Cytokine array expression analysis

Human Proteome Profiler XL Oncology array were purchased from R&D Systems. Primary MSC (0.25 × 10^6^) were transduced with PGC1a-KD virus or control-KD virus for 72 h. Media was changed and then harvested from these cultures after 24 h. Differential expression of secreted proteins was analysed according to the manufacturer’s instructions. Cytokine membranes were analysed using a BioRad Gel DocXR+ and quantified using ImageJ [45].

### Glucose uptake and consumption assays

Primary MSC (0.25 × 10^6^) were treated with melanoma conditioned media or control media for 48 h. Primary MSC (0.25 × 10^6^) were treated PGC1a-KD virus or control-KD virus for 72 h. Promega Glucose Uptake Assay was performed on treated cells and Promega Glucose-Glo Assay was performed on media from treated cells according to manufacturer’s instructions.

### Amplex Red assay

Amplex Red assay (ThermoFisher) was used to specifically measure the H_2_O_2_ levels generated. This reaction was carried out per the manufacturer’s specifications. Melanoma cells was plated on a black 96-well plate with a transparent base. Fluorescence was measured using the FLUOstar Omega microplate reader (BMG LABTECH, Ortenberg, Germany). A hydrogen peroxide standard curve was performed to determine the concentration of H_2_O_2_ levels generated by melanoma cells.

### Statistical analysis

Statistics will be generated using GrapPad Prism5 software (GraphPad, San Diego, USA). The Mann–Whitney *U* test will be used to compare test groups, with *p* < 0.05 to be considered statistically significant. Results are the mean ± standard deviation of four or more independent experiments.

## Results

### MSC migrate towards melanoma cells in vivo

**Fig. 1:**
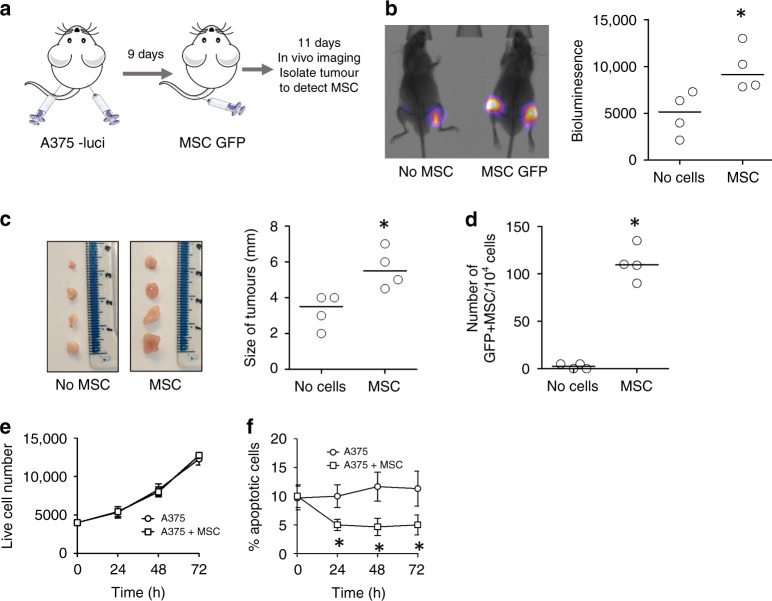
MSCs migrate towards melanoma cells. **a** Schematic diagram of experimental design. A375 melanoma cells were transduced with lentivirus containing a luciferase tag and then injected subcutaneously into 4 NSG mice for 9 days. After 9 days transduced MSCs (with rLV.EF1.AcGFP-Mem9 lentivirus) where injected intravenously into the tail vein of NSG mice for 11 days. **b** At day 20, mice were imaged for melanoma engraftment under anaesthesia and densitometry of the bioluminescent levels were calculated in control (no MSC) and test (MSC) mice. **c** Mice were sacrificed, and the tumours were excised. Size of tumour was measured in control (no MSC) and test (MSC) mice. **d** Tumour was digested and the cells were analysed for GFP fluorescence and corresponding number of GFP+ MSC in the tumour. *n* = 4 *p < 0.05. **e** A375-GFP (4 × 10^4^ cells) were cultured with MSC (0.25 × 10^5^) for 24–72 h. At each time point cells were removed from culture by trypsin and counted using the GFP to distinguish between MSC and melanoma cells. **f** A375-GFP (4 × 10^4^ cells) were cultured with MSC (0.25 × 10^5^) for 24–72 h. At each time point cells were removed from culture by trypsin and annexin V staining was performed and expressed as % apoptotic cells.

To assess the migratory effect of MSCs, conditioned media from A375 melanoma cells and control RPMI media were placed at the bottom of transwell plates, with MSCs seeded at the top. After 48 h co-culture the mean MSC cell count was significantly higher in conditioned media transwell compared to control media transwell (Supplementary Fig. 1). To determine if this migratory effect of MSCs is observed in vivo, an NSG xenograft model was used. Luciferase-tagged A375 melanoma cells were subcutaneously injected into the flanks of NSG mice (at day 1). At day 9, GFP labelled bone marrow derived MSCs were injected intravenously into the tail vein (Fig. 1a). At day 20, bioluminescence in vivo imaging showed that mice injected with both melanoma and MSC had greater tumour burden compared to melanoma injected alone (Fig. 1b). On day 20, mice were sacrificed and the tumours were excised. Visual measurements of the tumour showed enhanced growth of melanoma with MSCs, compared to the control (Fig. 1c). Next, these tumours were then digested, and single cells were analysed for GFP fluorescence. Analysis showed that GFP expressing cells were detected in the tumour of mice injected with MSC-GFP (Fig. 1d), suggesting that the MSC-GFP cells injected into the tail vein migrate towards and infiltrate the malignant tissue. To determine if MSC enhance tumour burden in vitro we performed proliferation and apoptotic assays. Results show that proliferation of A375 was not significantly different when cultured with MSC compared to media only (Fig. 1e). However, the number of A375 apoptotic cells, as measured by annexin V staining, was significantly less when cultured with MSC (Fig. 1f).

### Melanoma cells induce mitochondrial biogenesis in MSC

**Fig. 2:**
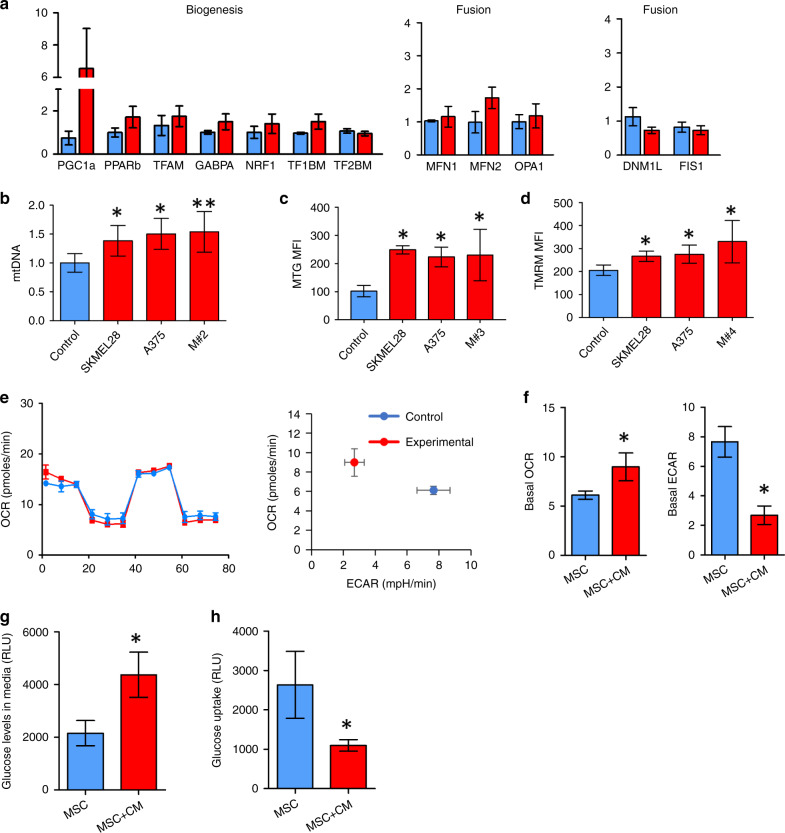
Melanoma cells induce mitochondrial biogenesis in MSCs. **a** MSCs were co-cultured with melanoma cells in transwells for 24 h. RNA from MSCs was isolated and analysed by qPCR. **b** MSCs were cultured for 48 h with control RPMI media and conditioned media (CM) from SKMEL28, A375, and specimen M#2. The DNA from MSCs were isolated and analysed by Taqman qPCR. **c** MSCs were cultured for 48 h with control RPMI media and CM. MSCs were stained with MitoTracker Green and analysed via flow cytometry. **d** MSCs were cultured for 48 h with control RPMI media and CM. MSCs were stained with TMRM and analysed via flow cytometry. **e**, **f** MSCs were cultured for 48 h with control RPMI media and CM. Seahorse extracellular flux analysis of the MSCs determined the influence of increased mitochondria on preferred mechanism of metabolism. *n* = 4, *p < 0.05. **g**, **h** MSCs were cultured for 48 h with control RPMI media and CM. Glucose consumption of MSC media and Glucose uptake by MSCs was determined. Data expressed as detected relative light units (RLU). *n* = 4, *p < 0.05.

We and others have shown that mitochondria can be transferred from non-malignant to malignant cells [22, 24, 26, 46–53]. Next, we wanted to assess the influence of melanoma cells on mitochondrial biogenesis and fission/fusion genes in MSCs in vitro. Human BMSCs were isolated and cultured with freshly harvested melanoma cells (isolation shown Supplementary Fig. 2A and B). RNA was extracted from both cell types and expression of mitochondrial genes was assessed. Upregulation of PGC-1α was observed in MSCs co-cultured with freshly harvested melanoma and melanoma cells lines (Fig. 2a and Supplementary Fig. 3).

As PGC-1α is the main regulator of mitochondrial biogenesis, we aimed to determine if upregulated of PGC-1α led to increased levels of mitochondria in MSCs. MSCs were cultured for 48 h with conditioned media from SKMEL28, A375, and clinical melanoma specimen M#2. Mitochondrial DNA (mtDNA) and genomic DNA were extracted and measured from the MSCs. Real-Time PCR showed increased mitochondrial DNA expression in MSCs cultured with conditioned media from SKMEL28 (Fig. 2b). The MSCs were also isolated from co-cultures and stained with Mitotracker Green (MTG). Flow cytometry analysis demonstrated increased mitochondrial content in MSCs cultured with conditioned media from melanoma cells (Fig. 2c). We subsequently used TMRM fluorescence to determine mitochondrial membrane potential in MSC cultured with media from melanoma cells. Flow cytometry analysis demonstrated increased mitochondrial membrane potential in all MSCs cultured with conditioned media from SKMEL28, A375 and M#4 compared to MSC monoculture (Fig. 2d). These results demonstrate that MSC have increased mitochondrial content and mitochondrial membrane potential when cultured with conditioned media from melanoma cells.

To understand the metabolic function of MSC when cultured with melanoma, we performed Seahorse extracellular flux assays. MSCs were cultured with conditioned media from specimen M#5 for 48 h and then analysed using XF Cell Mito Stress Test Kit. Results showed no changes in oxygen consumption rate (OCR) between MSC cultured alone and MSC cultured with melanoma condition media (Fig. 2e and f). However, extracellular acidification rate (ECAR) was significantly lower in the melanoma treated MSC compared to untreated MSC (Fig. 2f). To determine if the observed change in MSC metabolism was reflected in glucose uptake and consumption we performed two assays. Data show the relative light units which represent glucose uptake and consumption (glucose levels detected in the media). A lower RLU value in the consumption assay represents more glucose consumption were a lower RLU value in the uptake assay represents less glucose uptake. Therefore the data show that MSC cultured with CM had a significantly lower glucose consumption and glucose up take compared to untreated MSC (Fig. 2g and h).

### Melanoma induced PGC-1α drives mitochondrial biogenesis in MSCs which supports melanoma growth in vivo

**Fig. 3:**
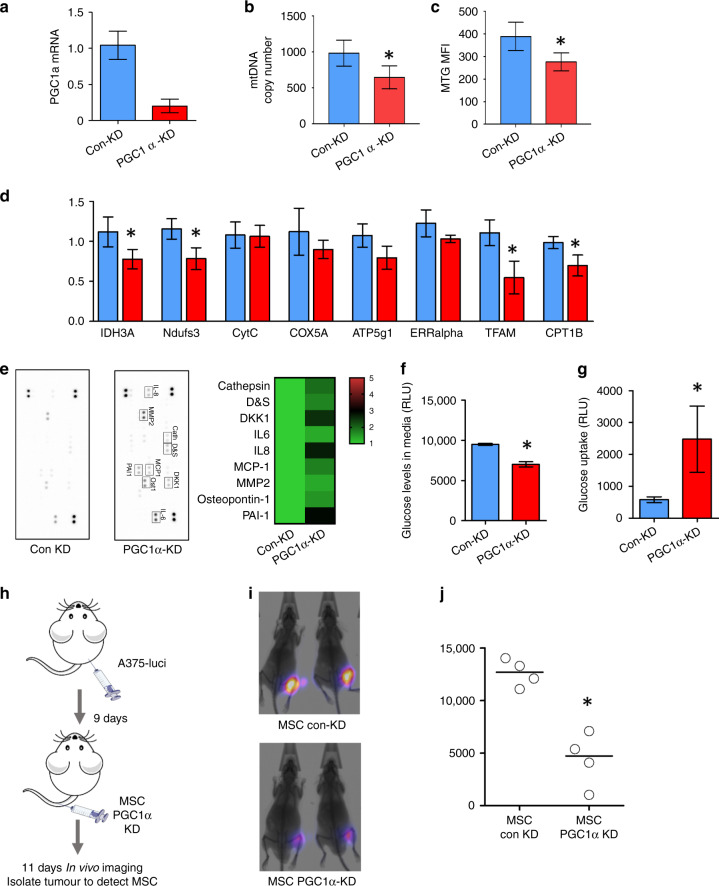
MSC-derived PGC1a is required for rapid melanoma tumour growth. **a** PGC-1α was knocked down (KD) in MSCs with shRNA lentivirus and confirmed with qPCR. **b** PGC-1α KD MSCs and control-KD MSCs were cultured with CM from SKMEL28. The DNA from MSCs were isolated and were analysed by Taqman qPCR for levels of mitochondrial DNA. **c** PGC-1α KD MSCs and control-KD MSCs were cultured with CM from SKMEL28. MSCs were stained with MitoTracker Green. **d** RNA was extracted from PGC-1α KD MSCs and control-KD MSCs and analysed by qPCR. **e** Cell-free supernatants from PGC-1α KD MSCs or control-KD MSCs (0.25 × 10^6^) were obtained and cytokine antibody array of each of the cell culture conditioned media using the Human Cytokine Proteome Profiler Array. Data represented as a heat map of detected changes in expressed cytokines. **f**, **g** PGC-1α KD MSCs or control-KD MSCs (0.25 × 10^6^) were analysed for glucose consumption (media) and glucose uptake (cells). Data expressed as detected relative light units (RLU). *n* = 4, *p < 0.05. **h** Schematic diagram of experimental design. A375 melanoma cells were transduced with a luciferase tag and injected subcutaneously into 10 NSG mice for 9 days. At day 9, PGC-1α KD MSCs and control-KD MSCs were injected intravenously into five mice each, respectively. **i** At day 20, mice were imaged for tumour growth. **j** Densitometry of the bioluminescent levels were assessed in control-KD and PGC-1α KD mice.

In order to determine if the mitochondrial biogenesis was driven by PGC-1α expression we knocked down (KD) PGC-1α in MSCs with shRNA lentivirus (Fig. 3a). PGC-1α KD MSCs and control-KD MSCs were cultured with conditioned media from SKMEL28 for 48 h. The MSCs were isolated and assessed for mitochondrial DNA and MTG. Real-Time PCR demonstrated reduced mitochondrial DNA expression in PGC-1α KD MSCs culture, compared to control-KD MSCs (Fig. 3b). In addition, MTG analysis revealed reduced mitochondrial content in PGC-1α KD MSCs compared to control-KD MSCs (Fig. 3c). Genes regulated by PGC-1α were also downregulated in MSCs with PGC1α KD (Fig. 3d). To ascertain the effect of PGC-1α KD in MSCs was analysed a panel of secretory factors using the Human Proteome Profiler Oncology Antibody Array. Protein array profiles of MSC with and without KD (Fig. 3e) showed a consistent upregulation of IL8, DKK1 and PAI-1 in PGC1α KD compared to control KD. To further characterise MSC with and without PGC1α KD, glucose uptake and consumption assays were performed as described in Fig. 2. Results show that reduced glucose concentration in media and increased glucose uptake in PGC1α KD cells (Fig. 3f and g), indicating glucose consumption was increased in PGC1α KD cells.

To demonstrate the functional consequence of PGC-1α driven mitochondrial biogenesis on melanoma growth in vivo, NSG mice were subcutaneously injected with A375 luciferase-labelled melanoma cells for 9 days to enable melanoma to engraftment (Fig. 3h) [22]. At day 9, PGC-1α KD MSCs, confirmed via qRT-PCR, and control-KD MSCs were injected into the tail vein. At day 20, bioluminescent in vivo imaging and quantification demonstrated reduced tumour growth in PGC-1α KD MSC injected mice compared to control-KD MSCs (Fig. 3i and j). Collectively these experiments show that PGC-1α driven mitochondrial biogenesis supports tumour growth.

### Mitochondrial transfer from MSCs to melanoma cells

**Fig. 4:**
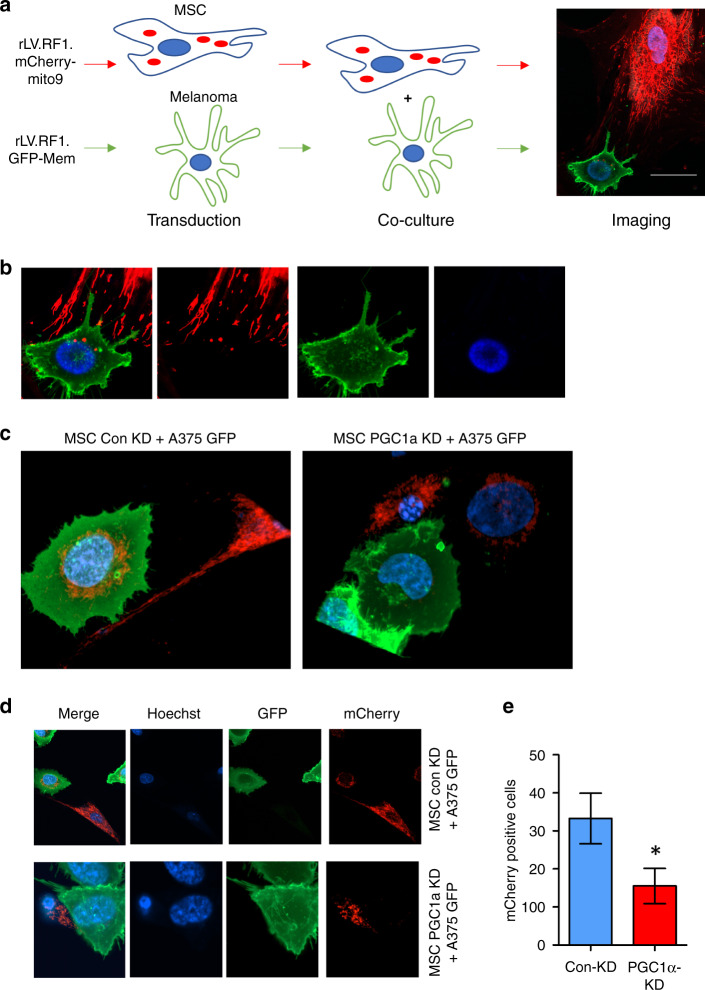
Imaging of mitochondrial transfer from human MSCs to human melanoma cells. **a** MSCs with their mitochondria labelled with Mito9 virus (red) and the A375 melanoma cells labelled with GFP membrane tag (green) were co-cultured on 24-well Ibidi microscopy plate for 24 h. Both cells were stained with Hoechst 33342 [66] to visualise the nucleus. **b** High resolution live microscopy of cultured cells. Scale bar = 20 µM. MSC labelled with Mito9 virus (red) were transduced with PGC-1α KD MSCs or control-KD and then cultured with A375-GFP membrane tag (green) for 24 h. Cells were stained with Hoechst 33342 [66] to visualise the nucleus. **c** Z-stack of representative images. **d**, **e** Quantification of A375-GFP cells containing mCherry fluorescence.

Next, we labelled MSC-derived mitochondria with mito9 virus (mCherry) and the A375 melanoma cells labelled with GFP membrane tag to visualise mitochondrial transfer from MSCs to melanoma cells (Fig. 4a). These two types of cell were then co-cultured on 24-well Ibidi microscopy plate for 24 h. Both cells were stained with Hoechst 33342 to visualise the nucleus. Images obtained from confocal live microscopy ultimately demonstrate transfer of whole mitochondria from MSCs to melanoma cells (Fig. 4b). To determine the effect of PGC-1α KD in MSC on mitochondria acquisition by melanoma cells labelled MSC were transduced with PGC-1α KD or control-KD virus and then cultured with A375 melanoma cells labelled with GFP. Images obtained from confocal live microscopy (Fig. 4c and d). Melanoma cells with mCherry fluorescence were counted between experiments and represented as mCherry positive cells (Fig. 4e).

### PGC-1α is upregulated by melanoma derived H_2_O_2_

**Fig. 5:**
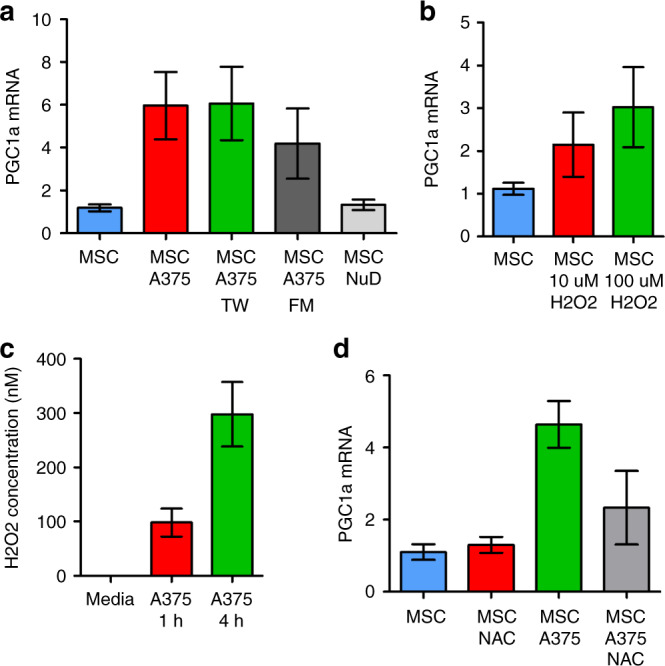
Melanoma derived H_2_O_2_ induces MSC PGC1α expression. **a** MSCs cultured with A375 in direct contact, in a transwell dish (TW), with A375 conditioned media filtered through a 0.22 µm filter (FM), or nutrient depleted media [54]. RNA extracted and PGC1α expression analysed by RT-PCR. **b** MSC were treated with 10 and 100 µM of H_2_O_2_ for 6 h. RNA extracted and PGC1α expression analysed by RT-PCR. **c** A375 were cultured for 1 and 4 h in fresh media. Media was collected and analysed for H_2_O_2_ using Amplex Red assay. **d** MSCs cultured with A375 in TW in the presence of 5 mM NAC for 24 h. RNA was extracted from MSC and PGC1α expression analysed by RT-PCR. All conditions except NuD induced PGC1a upregulation (**a**), suggesting that secreted factors were responsible for PGC1α upregulation in MSC. Others have shown that ROS can induce PGC1α in various cell types, therefore MSCs were cultured in the presence of H_2_O_2._ Both 10 and 100 µM induced PGC1α mRNA expression in MSCs. To determine if melanoma can produce H_2_O_2_ we use the Amplex Red assay. Melanoma produced detectable levels of H_2_O_2_ at 1 and 4 h post incubation. Finally, to determine if melanoma derived H_2_O_2_ is responsible for induced PGC1α mRNA expression in MSCs, H_2_O_2_ was quenched with the ROS scavenger N-acetyl-cysteine (NAC). PGC1α mRNA induced expression was inhibited in A375 cocultures in the presence of NAC. These data show that melanoma derived H_2_O_2_ induces MSC PGC1α expression.

To determine what melanoma-derived factors were involved in the induction of mitochondrial biogenesis, MSCs were treated with A375 in direct contact, A375 in a transwell dish (TW). A375 conditioned media filtered through a 0.22 µm filter (FM), as well as nutrient depleted media [54]. All conditions except NuD induced PGC1a upregulation (Fig. 5a), suggesting that secreted factors were responsible for PGC1α upregulation in MSC. Others have shown that ROS can induce PGC1α in various cell types, therefore MSCs were cultured in the presence of H_2_O_2._ Both 10 and 100 µM induced PGC1α mRNA expression in MSCs. To determine if melanoma can produce H_2_O_2_ we use the Amplex Red assay. Melanoma produced detectable levels of H_2_O_2_ at 1 and 4 h post incubation. Finally, to determine if melanoma derived H_2_O_2_ is responsible for induced PGC1α mRNA expression in MSCs, H_2_O_2_ was quenched with the ROS scavenger N-acetyl-cysteine (NAC). PGC1α mRNA induced expression was inhibited in A375 cocultures in the presence of NAC. These data show that melanoma derived H_2_O_2_ induces MSC PGC1α expression.

## Discussion

In this study we report that melanoma proliferation is enhanced by acquiring mitochondria from tumour supporting MSC. This process facilitates a shift from glycolytic metabolism towards oxidative phosphorylation. Furthermore, we have shown that bone marrow derived MSCs migrate to melanoma tumour, where mitochondrial biogenesis is stimulated in a PGC1α-dependent mechanism. The inhibition of PGC1α in MSC reduces mitochondrial transfer from MSC to melanoma. Overall, these results provide new insights into the metabolic changes in the melanoma tumour microenvironment.

Mitochondrial transfer has been described in many tumour types as well as under stressed, non-malignant conditions [28, 45]. Moreover, Dong *et al*. have previously shown that mtDNA transfers to mouse melanoma cells in vivo [55]. Our work shows that human melanoma cells can also acquire mtDNA from the tumour microenvironment suggesting this is not species-specific. The identification of the source of the mitochondria that are transferred to the tumour cells is important. Many studies have shown that mitochondria are transferred from MSCs. Where mechanisms of mitochondrial transfer (tunnelling nanotubules (TNTs), microvesicles and gap junctions) have been investigated previously [56], we re-demonstrate TNT mediated mitochondrial transfer between MSCs to melanoma cells (Fig. 4) as final step of mitochondrial transfer, allowing us to focus on our main aim of mitochondrial biogenesis in MSCs. In line with previous literature, we highlight need for further research into whether these transfer mechanisms occur simultaneously or separately, and pathways involved in each transfer mechanism [57]. In melanoma, others have shown that MSCs enhance tumour initiation and growth [58–61]. Our results demonstrate that melanoma cells have strong migratory signals which can attract BMSCs to the site of the tumour. Subsequently, the tumour signals to the MSCs to increase mitochondrial biogenesis and corresponding mitochondrial transfer to enhance tumour initiation and growth.

The data highlights the increased expression of the PGC1α in MSC when cultured with SKMCL28 and primary melanoma cells. It is interesting to note that we also see a slight increase in MFN2 which is involved in mitochondrial fusion [62]. However, no other genes associated with mitochondrial fusion or fission are upregulated in MSC cultured with melanoma. Where it has been shown that PGC1α is one of the first genes upregulated for increased mitochondrial biogenesis to occur in response to stress [63], the differential expression of other biogenesis transcriptional factors between MSCs co-cultured with SKMCL28 cell line and primary melanoma may be due to the timing of the experiment. Moreover other genes associated with this response like TFAM and NRF1 as well as genes involved in fusion and fission are not necessarily upregulated in response to stress [64]. Therefore, the expression of genes responsible for the transcription of the mitochondrial genes involved in mitochondrial biogenesis in the context of MSC warrants further investigation as the mechanisms for this are unclear. As it is beyond the scope of this paper, we publish this paper to demonstrate the potential of PGC1α derived mitochondrial biogenesis in MSCs as a therapeutic target and thus pave way for further research into PGC1α derived mitochondrial biogenesis in MSCs including the impact of PGC1α activity on general activity of MSCs, comparison of transcriptional profiles of activated MSCs and PGC1α KD MSCs, and melanoma-derived factors inducing mitochondrial biogenesis in MSCs.

The dynamic nature of OXPHOS and glycolysis in melanoma suggests metabolic plasticity, in that it is not fixed during carcinogenesis and that melanoma in fact has a ‘hybrid’ of OXPHOS/glycolysis phenotype [16, 65]. Our results confirm this plasticity, where we observed a significant decrease in ECAR when melanoma cells are cultured with MSCs and a complementary trend towards increased OXPHOS. We did not observe a significant increase in OCR, as expected, attributed to the shorter culture time of 48 h and would expect statistically significant increase in OXPHOS at 72-h timepoint or more.

In conclusion, we report that MSCs migrate to melanoma and are stimulated to produce mitochondria via PGC-1α. Without PGC-1α mitochondrial biogenesis is inhibited and mitochondrial trafficking is subsequently reduced from MSC to melanoma. These experiments together have increased our understanding of the pathophysiology of the disease, in terms of mitochondrial dynamics, suggest mitochondrial biogenesis in tumour supporting MSC as a potential therapeutic target that warrants further research.

## Supplementary information

Supplementary figures

## Data Availability

No datasets were generated or analysed during this study and all data is available upon request from the corresponding author.
