# Supplementary material for: PGC-1α induced mitochondrial biogenesis in stromal cells underpins mitochondrial transfer to melanoma
Source: Br J Cancer. 2022 Mar 26;127(1):69–78. doi: 10.1038/s41416-022-01783-w (PMC9276678; doi:10.1038/s41416-022-01783-w)
Supplement: Supplementary file 1 — Supplementary figures [file 41416_2022_1783_MOESM1_ESM.pptx]

## Slide 1
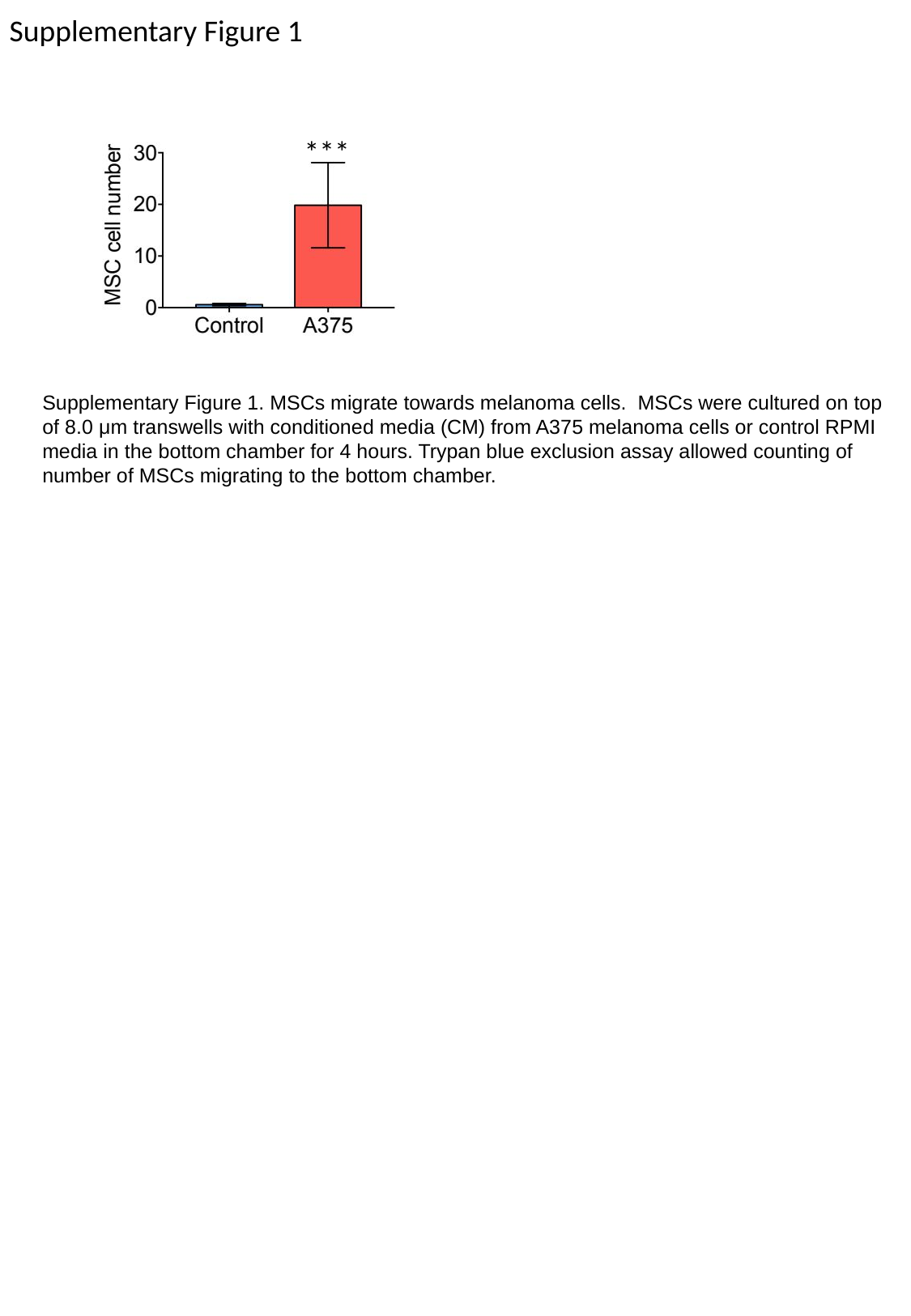

Supplementary Figure 1
***
Supplementary Figure 1. MSCs migrate towards melanoma cells. MSCs were cultured on top of 8.0 μm transwells with conditioned media (CM) from A375 melanoma cells or control RPMI media in the bottom chamber for 4 hours. Trypan blue exclusion assay allowed counting of number of MSCs migrating to the bottom chamber.

## Slide 2
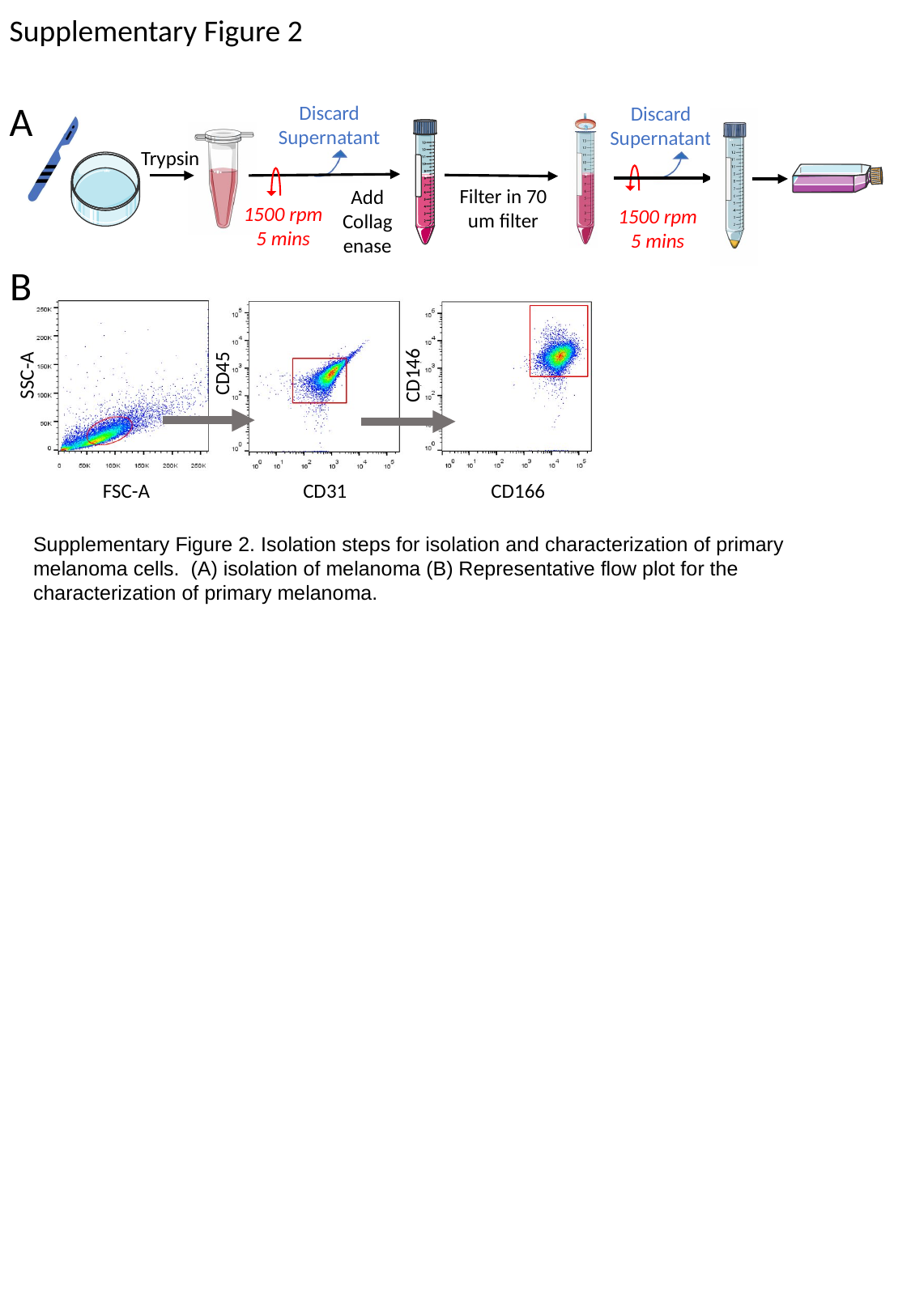

Supplementary Figure 2
A
Discard Supernatant
Discard Supernatant
Trypsin
Filter in 70 um filter
Add Collagenase
1500 rpm 5 mins
1500 rpm 5 mins
B
CD45
SSC-A
CD146
FSC-A
CD31
CD166
Supplementary Figure 2. Isolation steps for isolation and characterization of primary melanoma cells. (A) isolation of melanoma (B) Representative flow plot for the characterization of primary melanoma.

## Slide 3
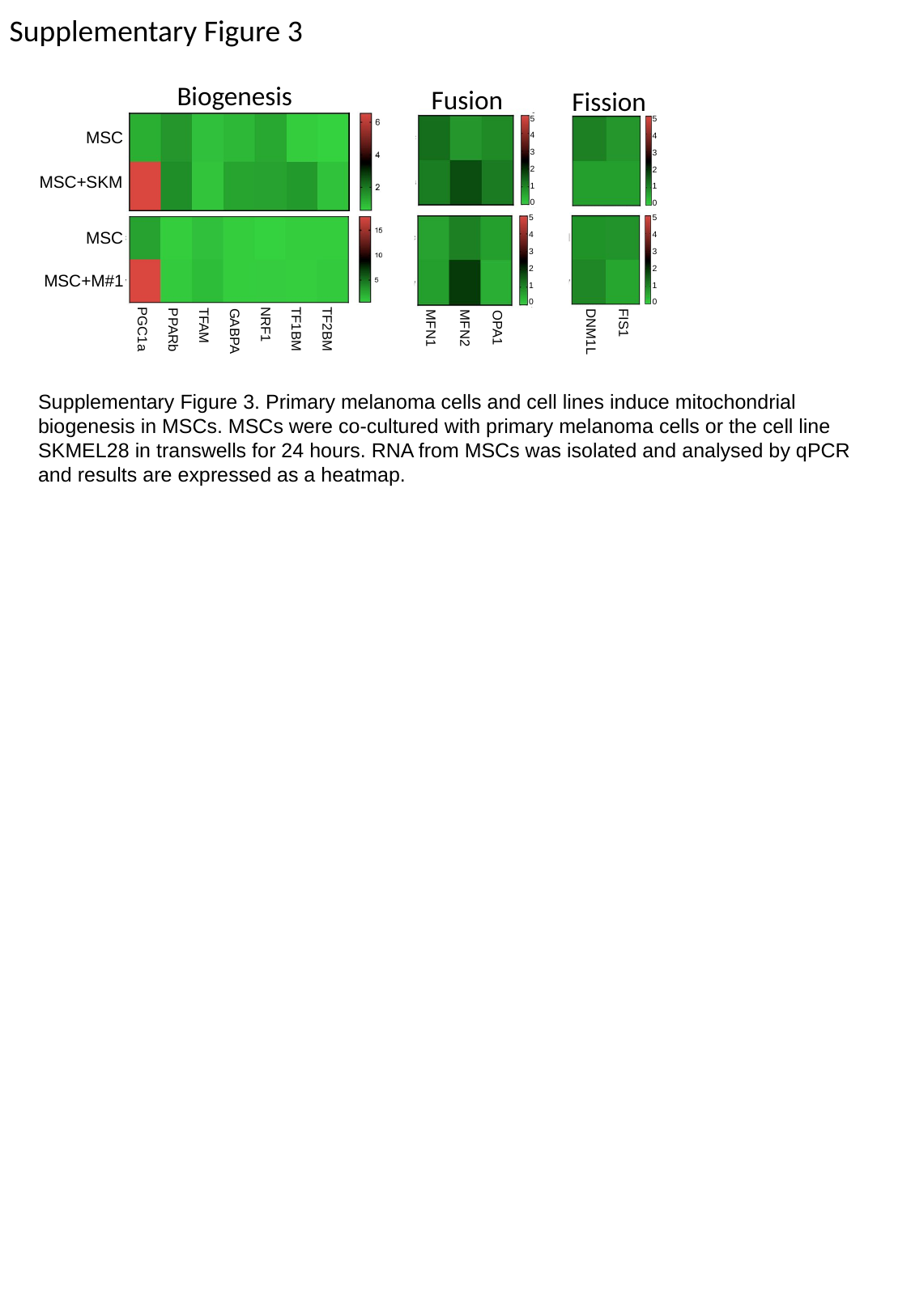

Supplementary Figure 3
Biogenesis
Fusion
Fission
5
4
3
2
1
0
5
4
3
2
1
0
MSC
MSC+SKM
5
4
3
2
1
0
5
4
3
2
1
0
MSC
MSC+M#1
FIS1
NRF1
TFAM
OPA1
MFN1
MFN2
PGC1a
TF1BM
TF2BM
PPARb
GABPA
DNM1L
Supplementary Figure 3. Primary melanoma cells and cell lines induce mitochondrial biogenesis in MSCs. MSCs were co-cultured with primary melanoma cells or the cell line SKMEL28 in transwells for 24 hours. RNA from MSCs was isolated and analysed by qPCR and results are expressed as a heatmap.
